# The 10th Anniversary of Nanomaterials—Recent Advances in Environmental Nanoscience and Nanotechnology

**DOI:** 10.3390/nano12060915

**Published:** 2022-03-10

**Authors:** Ioannis V. Yentekakis

**Affiliations:** 1 Laboratory of Physical Chemistry & Chemical Processes, School of Chemical and Environmental Engineering, Technical University of Crete (TUC), 73100 Chania, Crete, Greece; yyentek@isc.tuc.gr; Tel.: +30-28210-37752; 2 Foundation for Research and Technology—Hellas/Institute of Geoenergy (FORTH/IG), Technical University of Crete, Building M1, University Campus, Acrotiri, 73100 Chania, Crete, Greece

## 1. Overview

As a result of the rapid growth of nanoscience and nanotechnology, including advanced methods of fabrication and characterization of nanostructured materials, great progress has been made in many fields of science, not least in environmental catalysis, energy production and sustainability [1,2,3,4,5,6,7,8].

Also known as “nano-catalysts” [1,2,3,4,5,6,7,8], they now play a leading role in environmental and energy science and engineering by providing innovative, cost-effective, and durable nanostructured materials with highly promising performance in the control of environmental pollutants, production of clean fuels and added-value chemicals, and circular-economy technologies [5,6,7,8,9,10,11,12,13,14,15,16,17,18,19,20,21,22].

Indeed, the rational design of materials, particularly at the nano-to-atom level, offers advantages and enables tailoring and fine-tuning of their critical points in catalysis textural, structural, physicochemical, and local surface chemistry properties, as well as the optimization of metal–metal and metal–support interactions, thus resulting in catalytic systems with outstanding activity and stability performance in numerous eco-friendly applications. These include, for example, emission-control catalysis, waste treatment, photocatalysis, bio-refinery, CO_2_ utilization and fuel cell applications, as well as hydrocarbon processing for H_2_, added-value chemicals and liquid fuels production [5,6,7,8,9,10,11,12,13,14,15,16,17,18,19,20,21,22,23,24,25]. 

Celebrating the 10th anniversary of *Nanomaterials*, the Editor-in-Chief, Prof. Dr. Shirley Chiang, and the members of the Editorial Office organized and led a series of Special Issues on key topics that cover and highlight the journal’s aims and scope as well as future scientific and technical perspectives of the area. The present SI entitled “Advances in Environmental Nanoscience and Nanotechnology” was one of them, which aimed to host significant advances in the title. 

In this context, this SI has succeeded in collecting five high-quality contributions, refs. [26,27,28,29,30], covering recent research progress in various sub-directions of the field. These contributions are briefly discussed below.

## 2. Special Issue’s Contribution and Highlights

The capture, utilization, and recycling of CO_2_ emissions pose major challenges today due to the urgent need for the mitigation of global warming through the greenhouse effect and the concomitant major climate changes. CO_2_ hydrogenation to produce renewable fuels (e.g., methane or methanol) is one of the most attractive alternatives for this target, which will contribute to a cleaner and more sustainable future. Notably, CO_2_ methanation, also known as the *Sabatier reaction*, becomes more attractive if hydrogen demands for CO_2_ methanation reaction can be provided via a water-splitting process based on solar- or wind-powered systems. This further expands the eco-friendly and sustainability feature of the CO_2_ methanation concept, which is described as the power-to-gas process and has attracted intense attention as a promising method for the storage of hydrogen, as well as overcoming the safety, storage, and transport difficulties in H_2_ managing. 

The above benefits of thermo-catalytic CO_2_ methanation reaction are highlighted in the review paper of Tsiotsias et al. [26], in which the recent literature on the subject is overviewed and analyzed, giving particular attention on advanced bimetallic, Ni-M (M = Fe, Co, Cu, Ru, Rh, Pt, Pd, Re)-based catalyst formulations aiming to enhance the reaction activity and selectivity towards CH_4_, especially in the low temperature region (ca. 200–350 °C). This thorough literature overview and analysis allowed the authors to conclude that although Ni is among the most active methanation catalysts, similarly to the very active Ru and Rh noble metals, it is being favored considering its low cost and high abundance in nature. However, there are some drawbacks of Ni as a methanation catalyst, such as insufficient low-temperature activity, low reducibility and a substantial propensity for nickel nanoparticle sintering. Literature results show that these inefficacies can be partly overcome via the incorporation of a second transition metal (e.g., Fe, Co) or a noble metal (e.g., Ru, Rh, Pt, Pd and Re) at low loading in Ni-based catalysts. In that way, the formation of several possible mixing nanoparticle (NP) structures, such as “*alloyed*” or “core-shell” NPs or even “*Janus*” heterostructures (closely located, and thus interacting, Ni and M individual particles), causes strong electronic metal-to-metal effects between the two active phases; thus, new high-performing and low-cost methanation catalysts can be obtained. For example, using Fe and Co as heteroatoms due to their similar size and electronic properties with the Ni atom allow for their easy dissolution into the Ni lattice, forming NiFe and NiCo alloyed particles, respectively. The specific composition of the formed alloys can lead to the optimization of CO_2_ methanation performance, especially in the case of NiFe alloys. The combined bimetallic catalysts can also offer additional advantages, such as higher stability, and sulfur-poisoning tolerance. On the other hand, using noble metals as heteroatoms in the Ni-M combination, an increase in the reducibility and dispersion of the Ni primary phase can be obtained, useful for the CO_2_ methanation reaction. Among noble metals, Rh and Pt can greatly enhance the catalytic activity for CO_2_ methanation when dissolved or deposited in small quantities on Ni-based catalysts. Finally, the authors assume that a trade-off between cost and catalytic activity for CO_2_ methanation catalysts can potentially be overcome via the development of bimetallic Ni-containing catalysts with an optimized Ni–dopant metal synergy.

In a highly comprehensive and interesting review, Al-Maqdi et al. [27] thoroughly analyze the recent research efforts on the development of enzyme-loaded flower-shaped nanomaterials, which are highly promising for biosensing, biocatalytic, and environmental applications. The authors explain how organic–inorganic hybrid nanoflowers (hNFs), a recently developed class of well-structured and well-oriented flower-like materials, through their unique structural and multifunctional properties, has gained intense interest and can be useful in several top technological applications. The structural attributes along with the surface-engineered functional entities of hNFs, such as size, shape, surface orientation, structural integrity, stability under reactive environments, enzyme stabilizing capability, and organic–inorganic ratio, all are key well-tailored properties and characteristics that can significantly contribute to and determine the hNFs applications. According to the authors, although the development of hNFs is still in its infancy, the rapid development of biotechnology in general and nanotechnology in particular makes hNFs a versatile platform for constructing enzyme-loaded/immobilized structures for a variety of applications, including detection and sensing-based, environmental- and sustainability-based, and biocatalytic and biotransformation applications. Readers of this work can find detailed information on many key issues related to the current advances in multifunctional NPCs that are particularly emphasized in the review, such as: (a) critical factors; (b) different metal/non-metal-based synthesizing processes (i.e., Cu-, Ca-, Mn-, Zn-, Co-, Fe-, multi-metal-, and non-metal-based hNFs); (c) their applications; and finally, (d) the interfacial mechanism involved in hNF development considering the three critical points, which are the combination of metal ions and organic matter, the petal formation, and the generation of hNFs. Bearing in mind that the subject is new, the review of Al-Maqdi et al. [24] can undoubtedly be a valuable source of expert information and therefore a useful tool for hNF designers and hNF application engineers.

Aiming at a low carbon footprint energy future, H_2_ production, storage and transport currently garners huge research interest. Besides the electrochemical water-splitting technology coupled with renewable energy sources for the high demand of electric power, hydrocarbons are the most important raw feedstock for hydrogen production. Although the primary focus is currently on the abundant-in-nature methane (as a key component of natural gas and biogas), the use of liquefied petroleum gas (LPG), mainly consisting of propane and butane, for hydrogen production has recently received increasing interest for various economic and availability reasons. Interestingly, two works on this theme were contributed to the present SI.

In the first paper, Ramantani et al. [28] systematically investigated the partial substitution of La by Sr and of Ni by Ru or Rh in the LaNiO_3_ perovskite structure to explore the effects of the nature and composition of the A- and B-sites of such substituted perovskites on the propane steam reforming (PSR) reaction towards syngas (H_2_+CO) production. Specifically, LaNiO_3_ (LN) and La_0.8_Sr_0.2_NiO_3_ (LSN) and noble metal-substituted LNM*_x_* and LSNM*_x_* (M = Ru, Rh; *x* = 0.01, 0.1) perovskite-based catalysts were studied. The authors found that the incorporation of Ru and Rh foreign cations in the A and/or B sites of the perovskite structure resulted in an increase in the specific surface area, a shift of XRD lines toward lower diffraction angles, and a decrease in the mean primary crystallite size of the parent material, which was more pronounced for materials with higher NM loading due to distortion of the perovskite structure. Under PSR reaction conditions, the in situ development of new phases including metallic Ni and La_2_O_2_CO_3_ on the LNM_x_, and metallic Ni, La_2_O_2_CO_3_, La_2_SrO*_x_*, La_2_O_3_, and SrCO_3_ on the LSNMx were resulted, which enhance the PSR activity of these catalysts. Although the LN catalyst exhibited higher primary activity compared to LSN, its PSR catalytic performance did not appreciably change upon partial substitution of Ni by Ru. In striking contrast, the authors demonstrated that partial substitution of Ni by Ru and especially Rh in the LSN perovskite resulted in the significant promotion of catalytic performance on the title reaction, further improved upon increasing the noble metal content from *x* = 0.01 to 0.1 in the LSNM*_x_* (M = Ru, Rh) perovskite matrix. Thus, the LSNRh_0.1_ catalyst found to perform extremely stable for at least 40 hours on stream due to the in situ formation of the La_2_O_2_CO_3_ phase, which facilitates carbon oxidation, preventing its accumulation. This catalyst was the best over all the samples studied, offering propane conversion of ca. 92% accompanied by a selectivity towards H_2_ as high as 97% at 600 °C; its pronounced catalytic performance was attributed to a synergy of well-dispersed Ru nanocrystallites with Ni^0^ species on the perovskite surface [25]. The above results once again confirm the value of using such multifunctional materials in significant catalytic reactions. The specialized properties of perovskites, such as multiple types of active centers, including surface oxygen vacancies, as well as labile lattice oxygen and mobile O^2−^ ions—properties that are easily adapted and optimized on a case-by-case basis by partial substitution of A and B sites with A’ and B’ alternatives—are particularly useful in catalysis, as, in turn, these properties can play multiple roles as reaction promoters and stabilizers of the catalytic systems [23,24,25].

In the second paper concerning the same reaction (PSR), reported by Kokka et al. [29], the catalytic performance of supported Ni catalysts with respect to the nature of the oxide supports used for the dispersion of Ni particles was investigated. The authors found that Ni was much more active when supported on ZrO_2_ or yttria-stabilized zirconia (YSZ: Y_2_O_3_-ZrO_2_) compared to TiO_2_, whereas Al_2_O_3−_ and CeO_2_-supported catalysts exhibited intermediate performance. The comparison of the PSR performance of the catalysts was based on the turnover frequency (TOF) kinetic measurements, which demonstrated that support-induced promotional effects on the TOF of C_3_H_8_ conversion can be more than *one order of magnitude* higher, following the order Ni/TiO_2_ < Ni/CeO_2_ < Ni/Al_2_O_3_ < Ni/YSZ < Ni/ZrO_2_. These intrinsic rate increases were accompanied by a parallel increase in the selectivity toward the intermediate methane produced. Conducting in situ FTIR experiments, it was demonstrated that CH_x_ species produced via the dissociative adsorption of propane are the key reaction intermediates. Then, CH_x_ hydrogenation to CH_4_ and/or conversion to formates, and eventually to CO, is favored over the most active Ni/ZrO_2_ catalyst. In addition, the optimal Ni/ZrO_2_ catalyst exhibited excellent stability for more than 30 h time-on-stream (TOS) and was therefore proposed by the authors as the most promising of the series for the PSR process for H_2_ production.

Finally, Chalmpes et al. [30] reported a new synthetic approach towards carbon materials, which additionally provides a way to obtain useful materials out of waste or disposed rocket propellants. The method is based on the hypergolic ignition of furfuryl alcohol by fuming nitric acid at ambient conditions, which lead to the fast, spontaneous, and exothermic formation of carbon nanosheets in two steps: (i) polymerization of furfuryl alcohol to poly (furfuryl alcohol), and (ii) in situ carbonization of the polymer by an internal temperature increment near its decomposition point. A variety of advanced characterization techniques, such as XRD, infrared spectroscopy (Raman/IR), UV–vis, XPS, and SEM/TEM/AFM microscopies, were used to analyze the structure and morphology of the obtained carbon nanosheets. The method also provides for the direct conversion of the released energy into useful work by either heating acetone to boiling or spinning the Crookes radiometer. The authors argue that in a broader sense, the furfuryl alcohol-fuming nitric acid system could be the basis for a future “carbon from rocket fuel” perspective, especially given the wealth of available rocket bipropellants, as well as the growing progress for new hypergolic fuels. For such a perspective, the authors conclude that a technical upgrade of the method, as well as the search for new hypergolic pairs that will provide even higher carbon yields, remain future challenges for large-scale safe application.

Considering all of the above, I believe that the present SI has collected high-impact works that highlight valuable specific topics and current research interests in the field of environmental nanoscience and nanotechnology, as well as crucial perspectives and promising implementations. I would like to sincerely thank all the authors and reviewers who, with their valuable participation, have contributed to the production of these high-quality and high-impact papers that add value and can encourage researchers in the field.

Finally, I would like to take the opportunity to encourage researchers to submit their best works in *Nanomaterials*, a journal that has already been established as a high value, readability, and impact one, very suitable to host high-quality works in the field of nanomaterials/nanotechnology. As Editor-in-Chief of the highly active “Environmental Nanoscience and Nanotechnology” section of the journal, I would kindly appreciate your contributions to this area as well.
